# Supplementary material for: Evaluation of Fecal Glucocorticoid Metabolite Levels in Response to a Change in Social and Handling Conditions in African Lions (Panthera leo bleyenberghi)
Source: Animals (Basel). 2021 Jun 24;11(7):1877. doi: 10.3390/ani11071877 (PMC8300219; doi:10.3390/ani11071877)
Supplement: Supplementary file 1 [file animals-11-01877-s001.zip › animals-1214871-supplementary.pdf]

Table S1. Concentrations of FCM of the samples analyzed in duplicate or triplicate to calculate intra and inter-assay coefficients of variation of the EIA kits. Bold numbers denote mean intra or inter-assay CV.

Intra-assay CV (EIA 1)					
FCM (ng/g)					CV (%)
Lecture 1	Lecture 2	Lecture 3	Mean	SD	
19.35	20.19	22.74	20.76	1.76	8.49
15.55	15.70	18.06	16.43	1.41	8.57
10.72	12.33	13.12	12.05	1.22	10.15
12.73	15.55	16.29	14.86	1.88	12.66
					**9.97**

Intra-assay CV (EIA 2)				
FCM (ng/g)				CV (%)
Lecture 1	Lecture 2	Mean	SD	
27.69	25.49	26.59	1.56	5.86
18.73	16.97	17.85	1.24	6.97
16.19	14.24	15.22	1.38	9.06
16.81	14.69	15.75	1.50	9.50
13.03	10.80	11.91	1.58	13.24
14.79	12.16	13.47	1.86	13.79
				**9.74**

Inter-assay CV				
FCM (ng/g)				CV (%)
Lecture EIA 1	Lecture EIA 2	Mean	SD	
23.16	24.06	23.61	0.64	2.70
27.65	29.85	28.75	1.56	5.41
21.48	23.84	22.66	1.67	7.36
28.14	25.29	26.71	2.02	7.55
15.66	11.21	13.43	3.15	23.43
10.65	15.76	13.20	3.62	27.40
				**12.31**

Abbreviations: CV, coefficient of variation; EIA, enzyme immunoassay; FCM, fecal cortisol metabolite; SD, standard deviation.

Table S2. Group (F1, F2, F3, M2) and individual mean (± SD) fecal cortisol metabolite (FCM) concentrations before (P1, P2) and after (P3) the death of the dominant male.

Individual/Group	Phase	FCM (ng/g)	Sample size
Female 1 (F1)	P1	19.60 ± 6.51	11
	P2	15.81 ± 5.44	7
	P3	13.80 ± 4.38	10
Female 2 (F2)	P1	18.02 ± 3.02	11
	P2	17.72 ± 5.32	7
	P3	17.76 ± 3.93	6
Female 3 (F3)	P1	20.72 ± 7.98	7
	P2	19.68 ± 4.12	8
	P3	14.37 ± 4.07	8
Male 1 (M1)	P1	22.00 ± 1.75	4
	P2	21.94 ± 8.80	12
	P3	-	-
Male 2 (M2)	P1	16.01 ± 4.71	15
	P2	15.80 ± 6.44	11
	P3	11.25 ± 2.49	13
GROUP (F1, F2, F3, M2)	P1	18.16 ± 5.60	44
	P2	17.15 ± 5.50	33
	P3	13.67 ± 4.15	37
